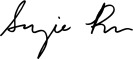# Introduction to Editorial Board Member: Professor Mark E. Davis

**DOI:** 10.1002/btm2.10056

**Published:** 2017-02-28

**Authors:** Suzie H. Pun

**Affiliations:** ^1^ Department of Bioengineering, University of Washington, Seattle, WA 98195.

In this issue, we would like to introduce our Editorial Board Member, Prof. Mark E. Davis. Prof. Davis is the Warren and Katharine Schlinger Professor of Chemical Engineering at the California Institute of Technology and a member of both the City of Hope Comprehensive Cancer Center and the UCLA Jonsson Comprehensive Cancer Center. He is one of the rare select individuals elected to all three United States National Academies (Engineering, Science and Medicine) as well as to the National Academy of Inventors for his major contributions to the fields of catalytic materials, biomaterials and drug delivery. He has also founded three biotechnology companies: Insert Therapeutics, Calando Pharmaceuticals and Avidity Biosciences.

Professor Davis earned his B.S. in Chemical Engineering from University Kentucky, where he attended on a full athletic scholarship. He continued his graduate education at the same institution, earning both his M.S. and Ph.D. degrees in Chemical Engineering. After graduation, he started his independent faculty position at the Virginia Polytechnic Institute & State University in 1981, where he was recognized with the NSF Presidential Young Investigator award (1985) as he quickly rose to full professor by 1989. During this time, his group was the first to report synthesis of a zeolite, a microporous crystalline material, with uniform pore sizes larger than 1 nm.^1^ For this previously unattainable achievement along with his other body of work in catalysis and reaction engineering, Professor Davis received the NSF Alan T. Waterman award in 1990 as the first engineer with this recognition.

Professor Davis joined the Chemical Engineering faculty at the California Institute of Technology (Caltech) in 1991. His group at Caltech continued to pioneer new innovations in catalysis, including the development of molecular sieves functionalized with organic molecules,^2^ further expansion of zeolite pore size,^3^ and demonstration of molecular imprinting of amorphous silica for shape‐selective catalysis.^4^

In 1995, Professor Davis expanded his research to biomaterials for cancer treatment in response to his wife's diagnosis and fight against breast cancer. Remarkably, within 10 years entering this field, Professor Davis led the development of two technologies that have since entered human clinical trials. One technology was a self‐assembling nanoparticle for nucleic acid delivery. This formulation was the first targeted siRNA nanoparticle formulation to enter into human clinical trials.^5^, ^6^ The second technology was a polymer‐drug conjugate using sugar‐based polymers to stabilize and deliver camptothecin, and anticancer agent.^7^, ^8^ This platform, is now being evaluated in phase II clinical trials by Cerulean Pharma. More recently, Professor Davis's group has been developing targeted formulations for drug delivery into the brain.^9^, ^10^

In addition to the aforementioned awards, Professor Davis has also been recognized with the Prince Asturias Award for Technical and Scientific Research that was presented to him by the King of Spain, the American Institute of Chemical Engineers (AIChE) Allan P. Colburn and Professional Progress Awards, the American Chemical Society's Ipatieff Prize and the E.V. Murphree, Elmer Gaden, and Gabor Somorjai Awards.

Professor Davis is a dedicated teacher and mentor to his students. He has authored two textbooks (one with his brother Robert J. Davis) that have been used broadly in chemical engineering curriculum. He has mentored over 60 graduate students and over 40 postdoctoral fellows in his laboratory, as well as hosting visiting scientists from around the world. His laboratory is a wonderfully diverse and inclusive environment for research.

Professor Davis's record of excellence extends beyond his scientific research and teaching. As an undergraduate track athlete at University of Kentucky, he received the Top Scholar Athlete award. He returned to competitive running after a long hiatus, and in 2011, won the 400‐m dash Gold Medal in his age division at the 19th World Masters Championship, as well as the 200‐m dash Bronze Medal. He was also on the two USA Gold Medal teams for the 4 × 100 m relay and the 4 × 400 m relay.

Working in Professor Davis's laboratory was an incredible experience. He led us by example to seek solutions to impactful, important problems. His weekly group coffee at the Red Door Cafe contributed to the healthy research environment in his lab, and lasting relationships formed in his group. His boundless scientific curiosity taught us to also be limitless as we sought potential solutions in our research.

**Figure 1:**
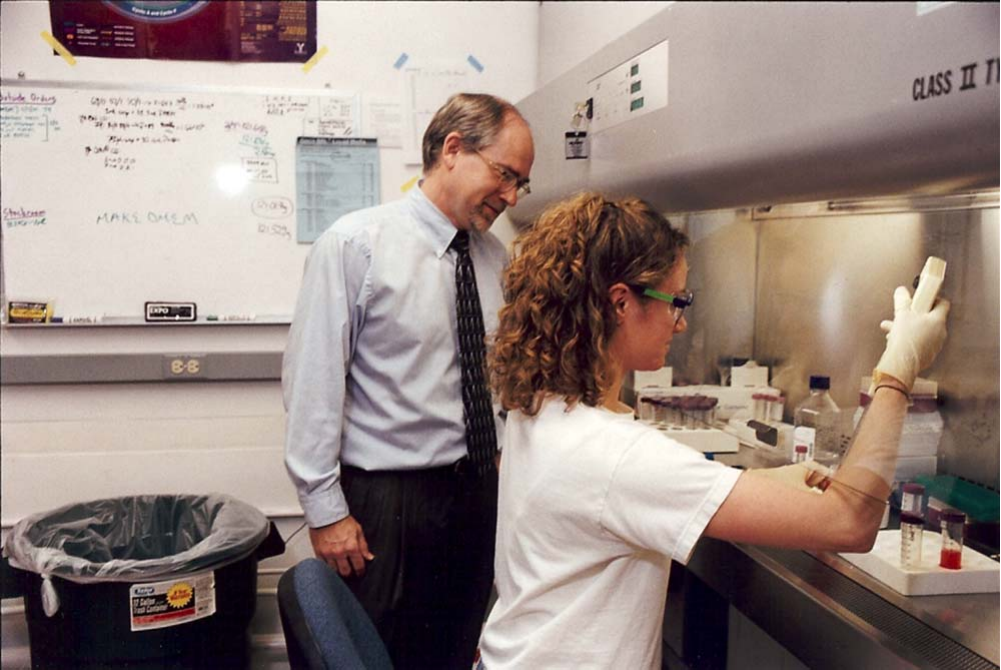
Mark E. Davis with Prof. Theresa M. Reineke when she was a postdoctoral fellow in his lab

**Figure 2:**
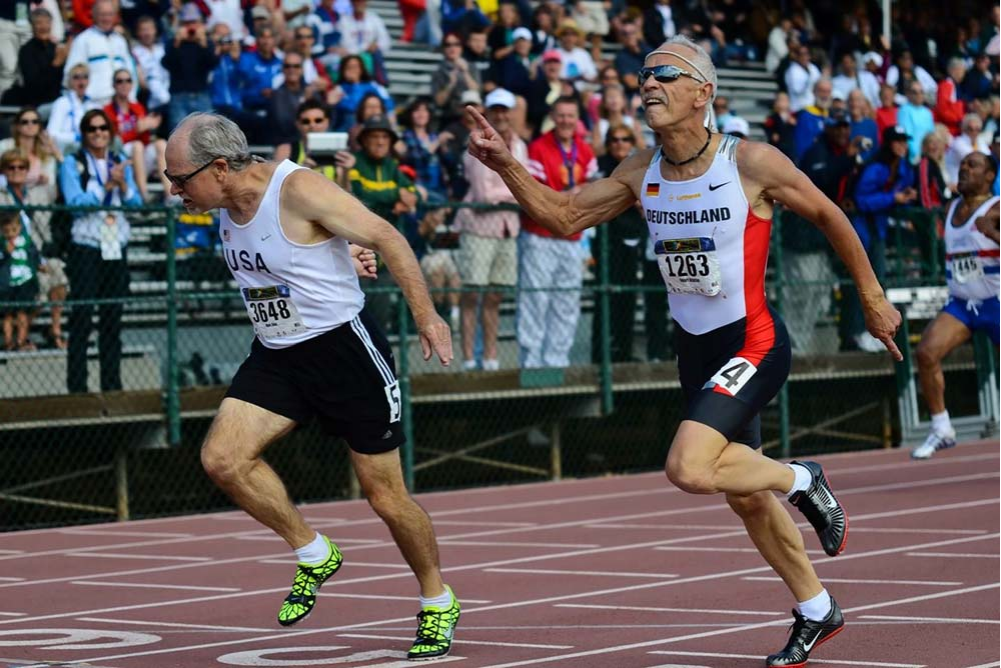
Mark E. Davis winning the 400‐m dash for his age division at the 19th World Masters Championship. Photo Credit: Darren Hall